# Balancing clinical relevance, legal boundaries, and technological solutions: a case-based analysis of secondary use of electronic health records in Sweden

**DOI:** 10.1016/j.esmorw.2025.100661

**Published:** 2025-12-15

**Authors:** Z. Dóczi, A. Valachis

**Affiliations:** 1 School of Behavioural, Social and Legal Sciences, Faculty of Humanities and Social Sciences, Örebro University, Örebro, Sweden; 2 Örebro University Hospital, Region Örebro County, Örebro, Sweden; 3 Department of Oncology, Faculty of Medicine and Health, Örebro University, Örebro, Sweden

**Keywords:** health law, secondary use of data, electronic health records, quality assurance, research, federated learning

## Abstract

**Background:**

The secondary use of electronic health records (EHRs) poses legal challenges, particularly when the responsibility for managing EHRs lies with local or regional authorities. This article presents a case-based analysis of the secondary use of EHR data in contexts where data privacy responsibilities are managed regionally in Sweden.

**Methods and results:**

Using two distinct purposes for the secondary use of the digital tool Patient Overview Breast Cancer: (i) assessing the uptake of new treatment strategies in a real-world setting for quality assurance, and (ii) evaluating the effectiveness of these strategies in specific patient subgroups with limited evidence for research purposes, the study explored the distinctions between research and quality assurance, the legal implications of each framework, and the potential role of federated learning as a privacy-preserving technological solution.

**Conclusions:**

Federated learning offers a promising approach to overcome legal and organizational barriers to secondary use of regional EHRs in Sweden, enabling scalable, clinically meaningful insights for cancer care. However, its effective implementation requires a unified national framework that balances personal integrity with patient safety, supported by regulatory sandboxes.

## Highlights

• Federated learning was assessed for scaling Swedish regional EHR data use.
• The main bottleneck for quality assurance was sharing personal data.
• In research, data sharing was less problematic when participants gave consent.

## Introduction

Electronic health records (EHRs) are a valuable source of real-world data, offering detailed and in-depth information on patients’ health and care.^1^ The secondary use of EHRs holds significant potential for research, particularly in clinical contexts where randomized evidence is impractical or unfeasible,^2^ and as a foundation for quality assurance in health care delivery.

Treatment strategies for patients with breast cancer have evolved significantly in recent years, both in curative and metastatic settings.^3^, ^4^, ^5^ New therapeutic options that often offer survival benefits at the cost of increased toxicity and resource demands are being developed. This makes it essential to better understand the real-world implications of these strategies. Key aspects requiring further exploration include the effectiveness of new treatments in broader populations than those typically included in clinical trials, optimal sequencing of therapeutic approaches, and the cost and resource implications of implementing new strategies in routine care.

The secondary use of EHRs for research and quality assurance also presents legal challenges, particularly in ensuring compliance with regulations such as the General Data Protection Regulation (GDPR), which governs data privacy, security, and governance in the European Union.^6^

Patient Overview Breast Cancer (IPÖ Breast Cancer) is a digital tool designed to compile and present comprehensive information about individual breast cancer patients. It visualizes a patient’s medical history and current status through a timeline, providing a clear and accessible overview. Relevant information regarding patient- and tumor-related characteristics, disease course, treatment strategies over time, their effectiveness, and associated toxicities is presented in a clear and structured format for each patient. Although it was primarily developed to support health care professionals in daily clinical practice, IPÖ also gathers structured data that are suitable for both research and quality assurance purposes. Legally, each health care region in Sweden is responsible for the implementation and operation of its own EHR systems, including IPÖ Breast Cancer.

Understanding how existing EHRs like IPÖ Breast Cancer can serve as sources for research datasets is both clinically and legally important. Because nationwide data sources are essential for research and quality assurance of cancer care, it is crucial to clarify how secondary data from regional health care providers may be shared at the national level from a legal perspective. In this study, we used IPÖ Breast Cancer as a case example to explore the extent to which secondary data from an EHR-based solution can be shared among regional health care providers, and to examine the types of data processing that are legally permitted.

## Material and methods

IPÖ Breast Cancer serves as a fit-for-purpose data source for the secondary use of data in both research and quality assurance, particularly in relation to the implementation of new therapeutic options in breast cancer care. To explore the legal aspects of secondary data use, we selected two clinically relevant case examples as theoretical use cases. For quality assurance (case 1), the theoretical objective was to assess the level and timing of uptake of newly reimbursed therapeutic options in clinical practice. For research purposes (case 2), the aim was to evaluate the effectiveness and real-world toxicity of these treatments in specific patient subgroups that are underrepresented or entirely excluded from pivotal clinical trials.

Both cases are interpreted in the applicable legal environment, where the primary and secondary use of EHRs are studied regarding data protection requirement (among other things) at Swedish regional health care providers.

Legal dogmatics is a well-established legal research approach focused on analyzing and interpreting existing legal rules and principles within a specific legal system. The content of the legal sources is interpreted to determine the meaning, purpose, and interrelation of the relevant legal provisions. With the help of the method, the applicable law is systematized, clarified, and conceptualized to reveal logical connections and principles understanding legal compliance of the two chosen case studies.

## Results

### Case study 1 implementation of new treatment strategies in breast cancer in clinical practice

#### Clinical perspective

Following the European Medical Agency approval and national reimbursement of a new breast cancer treatment, there is no structured mechanism in place for nationwide data collection to assess its implementation, including uptake levels, clinical effectiveness, and associated toxicities. This is particularly important given that many new treatment strategies, although potentially effective, are also associated with increased risks of both short- and long-term toxicity, financial burden, and greater demands on health care resources and costs.^7^^,^^8^

#### Legal analysis.

##### Purpose and ground

IPÖ Breast Cancer data is electronic health data, i.e. patient data. The legal purpose of case study 1 is quality assurance. Therefore, the legal ground of such data sharing among regional health care providers falls clearly under the Patient Data Act (2008:355).^9^ The Patient Data Act governs, among other things, the ability of health care professionals involved in a patient’s care to access the medical records required for treatment, even if those were created by a different health care organization.

##### Primary use

Quality assurance is traditionally considered as secondary use of health data. However, the technical setup in case study 1 raises the question of whether such systems could also have implications for primary use, namely, the real-time processing of EHRs in case of treating a certain patient. Although it is a technical possibility, it is not allowed to use the real-time personal data of a patient when providing health care to another patient at the same health care provider, through neither direct nor indirect access. This is because the processing of patient data from several patients for the care of another individual patient is not currently a particular permitted purpose according to the Patient Data Act (2008:355). Therefore, the potential primary-use application of this technical setup must be excluded.

Health care personnel are allowed to process patient data only when necessary to provide care to the specific patient whose data are being processed, and the person accessing the data must be directly involved in that patient’s care. These legal conditions are not met when data from one patient are used to inform the care of another, and such processing is therefore not permitted.^10^

##### Secondary use

The key issue is to understand whether it is legally possible to share personal information among regional health care providers for the quality assurance purposes in case study 1.

The prohibition as per Article 22 of the GDPR^11^ on automated individual decision-making including profiling is not applicable in a system merely established for medical reasons, i.e. assisting decision-making in diagnostics or further measures. If such a system is a part of a medical assessment or measure, this fact shall be documented in the health record of the patient, or it shall be added to it.^9^

Regional health care providers may jointly intend to utilize federated machine learning methods establishing such a system. Federated machine learning refers to a method where regional health care providers jointly train a machine learning model with no central data collection or pooling.^12^ The technology is one of the most common types of decentralized artificial intelligence. To develop such a system, local data sets that include patient data are required from each participating regional health care provider. The core idea is that there should be no transfer of personal data between the parties, i.e. regional health care providers do not share or gain access to any patient data processed at the other regional health care provider.

A risk with this method is that the local model, through training via iterations, may recreate patient data included in local training data. Another less probable yet possible risk is that via the model, a regional health care provider intentionally prepares access to the training data of another regional health care provider. The Swedish Authority for Privacy Protection noted two types of such attacks, namely membership inference attack and model inversion attack.^13^ The feasibility of such attacks is relatively low given the common interest, yet it cannot be ruled out completely. Therefore, the Swedish Authority for Privacy Protection does not exclude federated machine learning used for quality assurance purposes in case such a tool is implemented to establish measures which result in better and safer health care.

The risk that parameters in a machine learning model may open the possibility of leaking patient data shall be analyzed in each individual case and setup.^14^ GDPR is solely applicable on personal data. The Patient Data Act (2008:355) provides safeguards to patient data, including EHRs. If no personal data are shared among regional health care providers, it is possible to establish such a system using federated machine learning for quality assurance purposes. Technically, a risk and consequence analysis shall rule out the data protection impact of such a system. The analysis shall assess what kind of risk-mitigating measures are appropriate to consider a certain data type that does not contain personal data, such as using a larger range of cohorts and taking away certain variables (e.g. the specific contact data with a health care provider). It is important to point out that pseudonymization is not considered an appropriate measure, because patient reidentification may be possible according to the meaning of GDPR. The same applies even for rare diseases. Such legal assessments shall be made in each individual case and setup.

### Case study 2 effectiveness of novel treatment strategies across patient subgroups

#### Clinical perspective

The approval of new therapeutic strategies in breast cancer is based on results from randomized controlled trials (RCTs), which are designed to ensure high internal validity. However, their external validity can be questioned, as these trials are conducted in tightly controlled research environments with strict inclusion and exclusion criteria.^15^

Despite this, the results of RCTs are often extrapolated to broader patient populations in real-world clinical settings. This is problematic, as there is limited evidence on how these treatments affect specific patient subgroups, such as older adults, individuals with impaired functional status, or those with tumors exhibiting distinct biological characteristics.

#### Legal analysis.

##### Purpose and ground

The purpose of such a system is to assist research where a certain regional health care provider is the entity responsible for research with the intention of producing research results based on scientific data backed by an appropriate approval from the Swedish Ethical Review Authority. IPÖ Breast Cancer data is electronic health data, i.e. patient data, which is subject to research.

##### Primary use

Not applicable, because clinical research is the sole purpose of case study 2. Research may be considered in primary use solely if it is directly connected to the patient’s treatment. However, EHRs are used primarily for care, and secondarily for any clinical research.^9^

##### Secondary use

Regional health care providers may choose to implement a joint study on EHR stored in IPÖ Breast Cancer using a federated machine learning model. Such research activity is defined by a shared intention to generate new knowledge through scientifically grounded hypotheses, aimed at broad dissemination, and thus cannot be classified as internal business development or quality assurance.

In case study 2, sharing personal data among regional health care providers is a less pivotal issue compared with case study 1. If regional health care providers jointly steer the means and objectives of personal data processing, e.g. they jointly design the research plan, they are to be considered as joint data controllers as per Article 26 of the GDPR.^11^ The fact that a single regional health care provider may be appointed to be responsible for training the algorithm does not affect the shared responsibility for personal data processing. Each regional health care provider is a data controller for the processing of the locally stored personal data in such a research study. However, they are jointly responsible for the data processed in the global model.

Individuals have the right to object to the processing of their personal data for research, unless the processing is necessary to perform a task of public interest. The Swedish government has established that research activities is a task of public interest within the meaning of the GDPR.^16^ It concerns all research trials approved by the Swedish Ethical Review Authority that process sensitive personal data, including health data.

It shall be noted that the appropriate approval from the Swedish Ethical Review Authority is no guarantee that regional health care providers disclose the electronic health data in question for research purposes. Regional health care providers shall scrutinize the inquires in a legal assessment that is conducted when deciding whether to disclose personal information, particularly in health care settings, to ensure that such disclosure would not cause harm to the individual or their close relatives.^17^ Such legal assessment is unnecessary in the case of a research participant actively consenting to participate in the research in question.^17^

## Discussion

The current analysis provides a legal perspective on how secondary data from regional EHR-based solutions can be shared at the national level for research and quality-assurance purposes. A gap in legal analyses complicates situations as those highlighted in our case studies, where regional protections on personal data conflict with the need for national data collection and analysis to fully realize the value of secondary data use.

There are two main legal compliance issues to consider when applying federated machine learning in the analyzed cases. The legal purpose of the systems must be clearly defined. Since IPÖ Breast Cancer data consist of EHRs, i.e. patient data, the second issue is to determine whether the system involves the processing of personal data under the GDPR and patient data under the Patient Data Act (2008:355).^9^^,^^11^

Case study 1 is an example of quality assurance. Federated machine learning is allowed among regional health care providers only if there is no personal data in the dataset. There are examples of risk-mitigating measures to ensure that datasets do not contain personal data, including privacy-preserving strategies such as keeping data behind each institution’s firewall,^18^ using encrypted models,^19^ and applying data anonymization techniques.^20^ A legal uncertainty emerges because assessments shall be made in each individual case and setup. Moreover, it remains unresolved if such a dataset is still of clinical relevance and fit-for-purpose. For instance, anonymizing a dataset may compromise the granularity needed to obtain clinically relevant information, e.g. if anonymization involves grouping patient ages into broad categories, it could hinder investigations of treatment effectiveness within specific age groups.

Case study 2 represents clinical research. Federated machine learning is allowed among regional health care providers even in the case of personal and patient data in the dataset. There is no legal uncertainty in the case of all research participants actively consenting to participate in the research and when it is approved by the Swedish Ethical Review Authority. In any setup where active consent from a research participant is missing, a formal inquiry to disclose research data must be submitted to each corresponding regional health care provider. Each inquiry should be legally evaluated on a case-by-case basis. Consequently, it cannot be ruled out that, in some instances, personal data may not be disclosed for research purposes. Such restrictions on data disclosure could potentially affect the robustness of the dataset, leading to incomplete or biased data. This in turn may compromise the clinical relevance and generalizability of research findings.

In patient data processing, it should be noted the purpose of quality assurance, and the purpose of research shall never overlap as per the applicable law, i.e. no patient data used for quality assurance shall be used directly for research purposes, and vice versa.

The European Health Data Space (EHDS) Regulation aims to establish a common framework for the use and exchanges of EHRs across the EU. Its provisions are applicable on a Member State level, not among regional health care providers within a certain Member State. However, it is hoped that EHDS may accelerate changes in domestic regulation.

### Conclusion

In this theoretical analysis, federated learning appears to offer a promising solution to overcome legal barriers associated with the secondary use of regional EHRs for both quality assurance and research purposes, though it also highlights several distinct issues that must be addressed. From a clinical perspective, the use of federated learning could enable the necessary scaling of datasets from regional to national levels, thereby enhancing the potential to generate clinically relevant insights on implementation of new therapeutic strategies in cancer care.

The decentralized nature of Sweden’s regional health care system illustrates the need for a unified understanding on how electronic health data may be accessed, shared, and processed by solutions such as federated learning. Regulatory sandboxes can facilitate collective learning about the legal and technical constraints of emerging solutions; however, they cannot replace a comprehensive statutory framework.^21^ Today’s legal framework primarily focuses on protecting personal integrity. However, the other key objective, namely patient safety, can be significantly enhanced through the proportionate use of available technical solutions.
